# Detection of Carious Lesions and Restorations Using Particle Swarm Optimization Algorithm

**DOI:** 10.1155/2016/3264545

**Published:** 2016-04-26

**Authors:** Mohammad Naebi, Eshaghali Saberi, Sirous Risbaf Fakour, Ahmad Naebi, Somayeh Hosseini Tabatabaei, Somayeh Ansari Moghadam, Elham Bozorgmehr, Nasim Davtalab Behnam, Hamidreza Azimi

**Affiliations:** ^1^School of Dentistry, Zahedan University of Medical Science, Zahedan, Iran; ^2^Oral and Dental Disease Research Center, Department of Endodontics, School of Dentistry, Zahedan University of Medical Science, Zahedan, Iran; ^3^Oral and Dental Disease Research Center, Department of Oral and Maxillofacial Surgery, Zahedan University of Medical Science, Zahedan, Iran; ^4^State Key Laboratory for Manufacturing Systems Engineering, Systems Institute, Xi'an Jiaotong University, Xi'an, China; ^5^Department of Operative Dentistry, Oral and Dental Disease Research Center, Zahedan University of Medical Science, Zahedan, Iran; ^6^Department of Periodontology, Oral and Dental Disease Research Center, Zahedan University of Medical Science, Zahedan, Iran; ^7^Department of Public Health, Oral and Dental Disease Research Center, Zahedan University of Medical Science, Zahedan, Iran; ^8^School of Dentistry, Tabriz University of Medical Science, Tabriz, Iran

## Abstract

*Background/Purpose.* In terms of the detection of tooth diagnosis, no intelligent detection has been done up till now. Dentists just look at images and then they can detect the diagnosis position in tooth based on their experiences. Using new technologies, scientists will implement detection and repair of tooth diagnosis intelligently. In this paper, we have introduced one intelligent method for detection using particle swarm optimization (PSO) and our mathematical formulation. This method was applied to 2D special images. Using developing of our method, we can detect tooth diagnosis for all of 2D and 3D images.* Materials and Methods*. In recent years, it is possible to implement intelligent processing of images by high efficiency optimization algorithms in many applications especially for detection of dental caries and restoration without human intervention. In the present work, we explain PSO algorithm with our detection formula for detection of dental caries and restoration. Also image processing helped us to implement our method. And to do so, pictures taken by digital radiography systems of tooth are used.* Results and Conclusion*. We implement some mathematics formula for fitness of PSO. Our results show that this method can detect dental caries and restoration in digital radiography pictures with the good convergence. In fact, the error rate of this method was 8%, so that it can be implemented for detection of dental caries and restoration. Using some parameters, it is possible that the error rate can be even reduced below 0.5%.

## 1. Introduction

Caries is a multifactorial disease that is induced by the interaction among three factors, tooth, microflora, and diet. Radiography is a useful technique for detecting carious lesions as the caries process causes demineralization of enamel and dentin. Lesion is observed in a diagnostic image as a radiolucent (darker) zone since the demineralized area of the tooth does not absorb as many X-ray photons as the unaffected portion. Radiography is a valuable supplement to a thorough clinical examination of the teeth for detecting caries lesions.

Various morphologic phenomena, such as pits and fissures, cervical burnout, and Mach band effect, and dental anomalies, such as hypoplastic pits and concavities produced by wear, can mimic the appearance of a carious lesion [1–3].

Image processing has evolved since 1964. It has led to creation of digital multispectral ground surface images used in agricultural and forestry sectors. From mid-1970s to 1980s, the medicine has been evolved by invention of Computerized Axial Tomography scanners (CAT scan) and magnetic resonance imagery (MRI). Printing industry is another potential user of this technology. In addition, since entering to entrainment world at the end of 1980s, digital image processing has been common in this industry. By appearance of machine vision, the world of industry has been constantly evolving by robots. Purity in production of food vessels and their mass production has led to spending more time and cost on this technology. The high detection rate of decay and restoration of tooth by dentist require the mechanized systems with high quality and detection rate. Through such mechanized systems, it is possible to control and communicate with data in optimal databank and compare them with valid values in real-time control units to provide the optimum and fastest machine vision systems [4].

**Figure 1:**
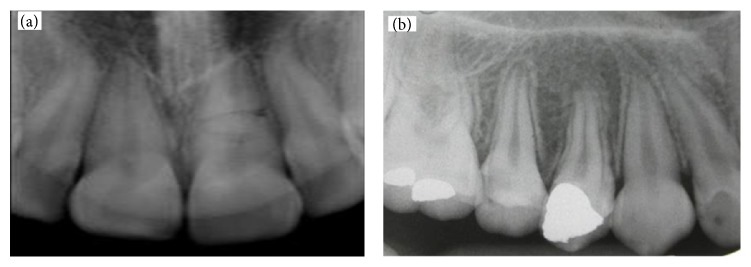
(a) Teeth with no decay and restoration. (b) Three teeth with decay and restoration.

Sometimes we need to complete the detection and restoration of teeth for a moment. Using the OCR frequent images of a tooth, it is possible to detect its decay and restoration in a fraction of a second. This is related to time of a qualitative problem, and the new system stops and detection process resumes once that problem is resolved [5]. The OCR pictures show the location of tooth in mouth. A dentist starts repairing the dental caries and restoration once he/she detects its location in mouth (Figure 1) [6].

In the second section, we review the previous research conducted in detection of dental caries and restoration and image processing. In addition, we introduce works related to detection with PSO or other algorithms in other fields of study. In the third section, we explain briefly our proposed method for detection of dental caries and restoration. In the fourth section, we present the results obtained after running our proposed model. Then, in the discussion section, we describe results of experiment and limitation of PSO with our formulation for 2D and 3D. Last section is related to conclusion.

## 2. Materials and Methods

Based on investigating previous works, no study was found on intelligent detection of dental caries and restoration using image processing and algorithms [5, 7]. No researcher has researched in this area.

**Figure 2:**
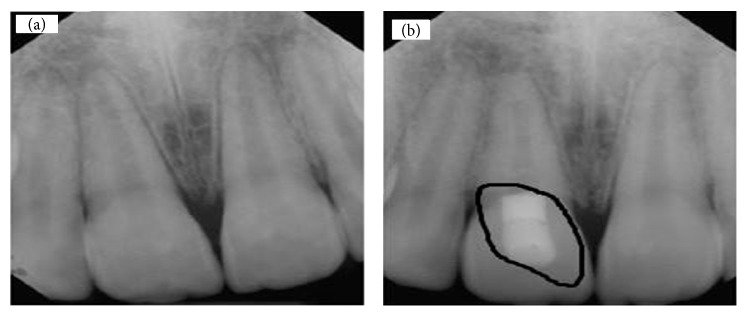
Detection of dental caries and restoration by dentist: (a) safe, (b) restoration.

All of the detections were done by dentist, as a dentist looks at picture and detects dental caries and restoration on basis of his own experiences.  Figure 2(a) illustrates detection of one spot of dental decay and restoration (Figure 2) [6].

**Figure 3:**
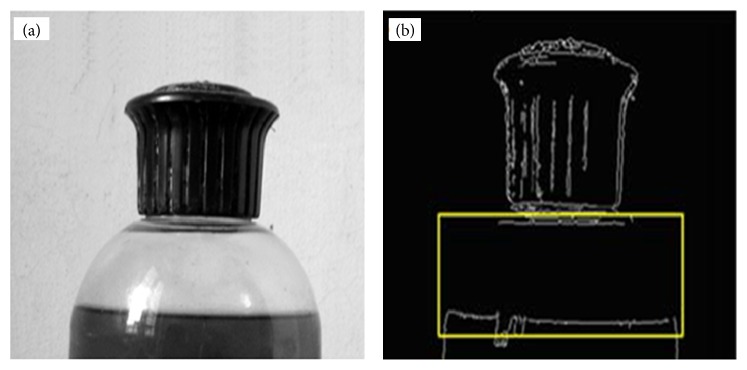
(a) Bottle of liquid. (b) Detection of surface level of liquid in bottle.

In another study, image processing was applied for detecting liquid level in bottle using some algorithms, rather than PSO, which are not either popular or efficient (Figure 3) [6]. Figure 3 indicates detection of liquid surface level using this algorithm [8, 9].

### 2.1. LOG Algorithm for Optimal Edge Recognition

The LOG algorithm shows that the location of edge occurrence is neither smooth nor thin. However, it is more efficient than previous method on low signal over noise. The steps of LOG algorithm can be summarized as follows [8, 9]: (a) convolving of image *I* by two-dimensional Gaussian function; (b) calculation of convolved image's Laplace; (c) edge pixel for passing from zero which is in *L*.

### 2.2. Algorithm Kani

Kani was assumed to be edge conditional to white Gaussian noise. The edge detector was considered with cannulation filter *f*, which distributes noise and location of edge. Here, the problem is to determine a filter that optimizes three criteria in recognition of a given edge. Algorithm Kani [10] is as follows:

(a) Reading image *I*.
(b) Convolving on one-dimensional Gaussian cover with *I*.
(c) Gaussian first derivative in the directions *x* and *y*.
(d) Convolved *I* with *G* along rows and bottom of columns to obtain *I*_*x*_ and *I*_*y*_, respectively.
(e) Convolved *I*_*x*_ with *G*_*x*_ to reach *I*_*x*_ and *I*_*y*_ with *G*_*y*_ to have *I*_*u*_.
(f) Finding results in any pixel.

## 3. Particle Swarm Optimization (PSO)

PSO algorithm was inspired from birds' behavior in their groups. This algorithm has fast and high convergence speed. Therefore, it has been efficiently used in static environments. Standard PSO algorithm has a population with *m* members, where each member is one potential solution in D-dimensional space of the problem [11, 12]. In the standard PSO algorithm, in the *t* iteration, the D-dimension of rate and situation of *i* member vary according to (1) and (2), respectively; *w*, *c*_1_, and *c*_2_ are nonnegative real constant parameters; and *r*_1_ and *r*_2_ are independent random numbers with uniform distribution in the range of 0 to 1. Consider

(1) Vidt+1=ωVidt+c1r1,idtPidt−Xidt+c2r2idtPgdt−Xidt,

(2) Xidt+1=Xidt+Vidt+1.

In FITNESS function, to obtain the threshold value for detecting the lesions in specific point (the center of lesions) based on the experience, we calculate an average of the selected color of the whole pixels of the image of human teeth. Hence, we do not take into account the points around the image of teeth which are not considered as the part of the human tooth. We multiply this average by 20% to obtain the threshold value for detecting the point where the lesions are formed around it. Because the quality of pictures may change by receivers, we alter this 20% to 1 to 3%, indeed 17% to 23% using *α* variable ((3) formula). By multiplying the specified value by *β* instead of *α*, the threshold value for the points surrounding pixels can be found (formula (4)). Consider

(3) Threshold  X=α×20100×∑i=1i=row ∑j=1j=columnpi,j;  pi=1,…,row,j=1,…,column≥a∑i=1i=row ∑j=1j=columncount=count+1;  pi=1,…,row,j=1,…,column≥a,

(4) Threshold  X  Around=β×20100×∑i=1i=row ∑j=1j=columnpi,j;  pi=1,…,row,j=1,…,column≥a∑i=1i=row ∑j=1j=columncount=count+1;  pi=1,…,row,j=1,…,column≥a.

The threshold value (Threshold *X*) for the central point is always less than the threshold value (ThresholdAround) for surrounding areas. Since this is the start point and end point of lesion, the threshold value will be always higher than that in other points, unless the lesion is improved.

For detecting the edges in four directions, we move pixel by pixel and review the threshold value of surrounding points, until a pixel is found with a threshold value higher than the threshold value of surrounding points. We save the spacing of these points to the central point (formula (5)). Then, we calculate the average of all points within a certain range around the central point (formula (6)). Consider
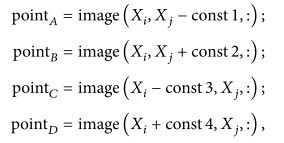
(5)

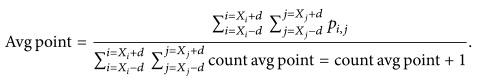
(6)


By investigating some conditions one can recognize that the central point can be the central point of lesion (formula (7)). The conditions are as follows: (1) the color of considered point should be lower than the threshold value. (2) The average of whole points within a certain range around the central point should be lower than the threshold value. (3) The difference between horizontal and vertical edges should be less than the threshold value separately, since this causes not going further away from the main edge of the teeth. Last part of FITNESS function is related to existence or nonexistence of preapical lesion in *X* point and around *X* point (7). Consider

(7) Exist_carious_lesion=1;Cx≤Xi,j≤Threshold  X  Around,Cx≤Avg point≤Threshold  X  Around,A−B||C−D≤Threshold  X  Around0;Other.

We explain some parameters below. The average points around *X* are Avg point. *P*, row, column, *a* and count are pixel (point) of picture, maximum row of picture, maximum column of picture, point of carious lesions and restoration teeth in picture, and number of selecting points for calculation. *P*_*i*,*j*_ is color value of image(*i*, *j*, 1) or image(*i*, *j*, 2) or image(*i*, *j*, 3). *X*_*i*,*j*_ is one point in place of image(*X*_*i*_, *X*_*j*_, 1,…, 3) and *X*_*i*_ is value of 1,…, row, *X*_*j*_ is value of 1,…, column, and *C*_*x*_ is constant value to avoid detection out of tooth.

## 4. Results

**Figure 4:**
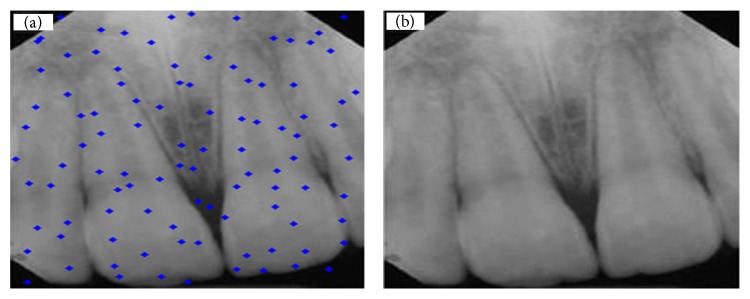
(a) Distribution of particles for detection of dental caries and restoration. (b) Teeth with no decay and restoration.

**Figure 5:**
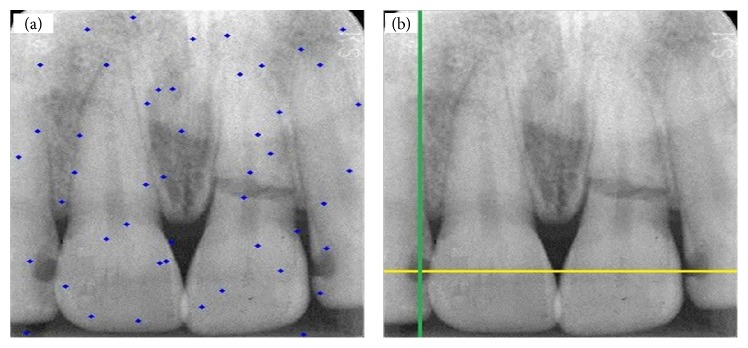
(a) Distribution of particles for detection of carious lesion and restoration. (b) Detection of one tooth with decay (right) depicted by green line crossed with yellow line.

**Figure 6:**
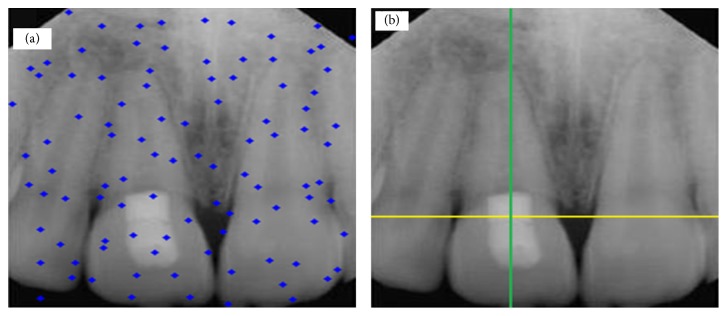
(a) Distribution of particles for detection of dental caries and restoration. (b) Detection of one tooth with decay and restoration, depicted by green line crossed with yellow line.

**Figure 7:**
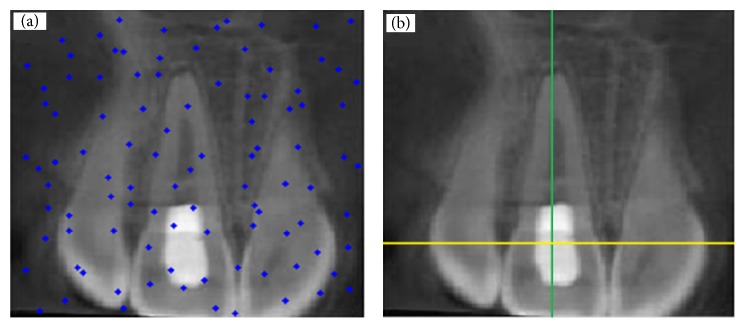
(a) Distribution of particles for detection of dental caries and restoration (b) and detection of one tooth with restoration, depicted by green line crossed with yellow line.

In this section, study results are presented.  Figure 4(b) shows a tooth with no decay and restoration, while the one in Figure 4(a) shows distribution of particles for detection of dental caries and restoration. Also two pictures were taken in the same mode. Figures 5, 6, and 7 show presence of decay and restoration in one tooth.

Through the experiments conducted in this research, first the particles are distributed throughout the pictures for diagnosis. Distribution of particles can be observed in (a) of Figures 4, 5, 6, and 7. Secondly, particles start to move in any picture. During running, PSO algorithm calculates value of transferring for any particle.

After running the algorithm, if the carious lesions or restoration is in teeth, PSO algorithm can recognize it and identify its location.  Figure 4 indicates a picture where there are no dental caries and restoration, while Figures 5, 6, and 7 illustrate that three other pictures have one case of dental caries and restoration. Our algorithm in Figure 4(b) does not show any dental caries and restoration. In comparison, the algorithm shows the place of dental caries and restoration for Figures 5(b), 6(b), and 7(b) through a green line crossing a yellow line.

**Figure 8:**
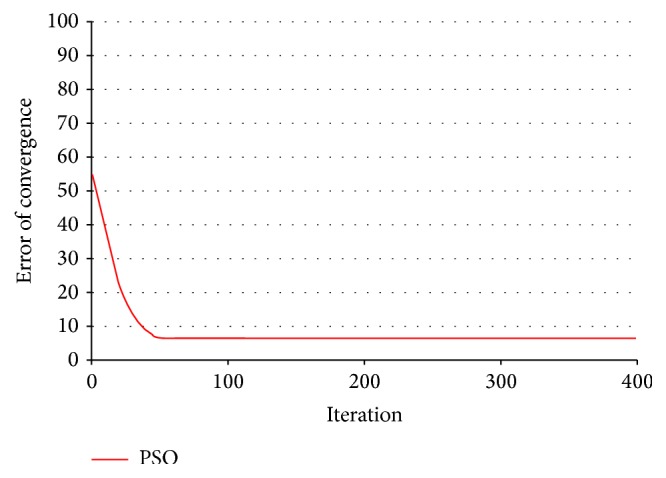
Converge of PSO during 400 steps.

The convergence of PSO is shown for 400 iterations in Figure 8. The algorithm converges very fast between 1 and 50 iterations. In other words, convergence occurs very fast. After that the rate of change is very slow for the next 400 iterations. The rate of change exactly is between 0.1 and 0.5.

## 5. Discussion

It has been shown that when six people without having any consultation with each other observe and interpret an X-ray radiographic stereotype, only 50% of them will have the same opinions; moreover, when radiographs are shown to a person at two different times, different interpretations are presented. Hence, it is recommended that, in order to interpret the radiographs, two independent people are used, and if there is disagreement between them, they should consult with each other to find an agreement, and if not so, one can ask a third person to offer his/her opinion; in this case, differences will be reduced from 50% to 25% [13].

Of mass particle swarm optimization (PSO) features, one can refer to the system memory in this system, in which the knowledge of proper solutions can be maintained by all the particles; in other words, in the algorithm of mass particle, every particle can benefit from its past information, while there are no such behaviors and features in other evolutionary algorithms. Also, in this algorithm, the populations are connected with each other and they solve their problem through the exchange of the information in a high speed convergence way. Using PSO algorithm with the help of image interpretation, the X-ray radiography processing error can be minimized. The differences in the interpretations of X-ray radiographies can be reduced from 25% to less than 8%, as well. Moreover, it saves time and increases interpretation accuracy. Our results show that this method can detect dental caries and restoration in digital radiography pictures. The error rate of this method was 8%, so that it can be implemented for detection of dental caries and restoration by dentist, since the error rate can be even reduced below 0.5%.

Therefore, we will do detection and repair of tooth diagnosis intelligently in the future. This method will come to help some special robots for our area with ability of dental lesions restoration without human intervention in the future. According to the authors of this paper, introducing this intelligent detecting lesions system in the future, as a result of designing the robots, will create a more prominent role, and developing of this intelligent system will support the clinicians in dental offices.

This method was used for 2D special images. At this time, it can find tooth lesions in Gary image. We also can apply color 2D image, because we just need to change detection value. But we did not apply it to 3D image for detection. We need to develop this method for 3D image. Then we will test on 3D images. If it has any problem, we will change some parameters to solve this problem. Also we need to test more different images that some different systems can take images of teeth. Our method is not a new algorithm. In fact, we use PSO algorithm and we have implemented the part of fitness by our formula.

## 6. Conclusion

In this paper, the image processing technique was introduced for some applications. One of these applications is the detection of dental caries and restoration. Therefore, we used both of image processing methods and particle swarm optimization (PSO) algorithm for detecting teeth decay and restoration. We implemented some mathematics formula for fitness of PSO. This idea helps us to solve this problem easily. Our results show that this method can detect dental caries and restoration in digital radiography pictures. The error rate was 8%, so that it is possible to use for detection of dental caries and restoration by dentist. Using the addition of some parameters, it is possible that the error rate can be even reduced below 0.5%. The convergence of this algorithm is good. But we can have very fast convergence using combinatorial optimization of some algorithms together. This method can be potentially used for detection of dental caries and restoration in the future. As the guideline for future works, the authors of this work recommend working on detection of dental caries and restoration using image processing and other popular algorithms.
